# Attracting dentists to the Swedish public dental services through the organisations’ websites

**DOI:** 10.2340/aos.v85.45661

**Published:** 2026-03-19

**Authors:** Cecilia Franzén

**Affiliations:** Faculty of Odontology, Malmö University, Malmö, Sweden

**Keywords:** Dentistry, organisational communication, recruitment, working conditions

## Abstract

**Objective:**

The recruitment and retention of dentists is important for quality dental care. The Swedish public dental service (PDS) suffers from staff shortages and high turnover due to perceived unfavourable working conditions that drive dentists towards the private dental sector. Websites can be a useful recruitment tool for organisations to communicate their mission, culture, and employee benefits. Therefore, this study aimed to investigate PDS organisations’ websites to identify how they present themselves to potential employees.

**Material and Methods:**

The data were collected from the websites of the 21 Swedish PDS organisations in June 2024. A thematic analysis was conducted using qualitative content analysis.

**Results:**

The findings show that PDS organisations appealed to dentists through emphasising their commitment to (1) fostering a sense of belonging within the workplace and (2) encouraging development and career growth, which are working conditions that can persuade employees to stay at an organisation. The organisations particularly appealed to newly graduated dentists through supporting the transition to dental practice.

**Conclusions:**

The PDS organisations emphasised favourable working conditions in their presentations of themselves as employers on their websites. Further research is needed on how effective the organisations’ websites are in attracting dentists and how dentists perceive the content on the websites in relation to actual workplace realities.

## Introduction

Human capital is valuable for organisations [1]. The knowledge and work experiences of dentists are significant for high quality dental care. In addition, the continuity of dentists at a workplace influences the quality of care because the frequent change of dentists might undermine the dentist–patient relationship [2]. Therefore, the recruitment and retention of dentists are key factors for patients’ oral health. The shortage of dentists can lead to long patient waiting lists and oral health inequalities due to limited access to dental care [3]. Furthermore, these shortages can increase the workload of the remaining dentists in the workplace. Although individual dentists differ in how they cope with their job demands, a heavy patient load and tight work schedules might generally contribute to perceived stress among dentists [4].

In Sweden, dental care is provided by both private dental care practitioners and by the public dental service (PDS), which is the national dental care run by all the 21 regions. The PDS is responsible for providing dental care for children and young adults up to the age of 19 years, although patients within this group can also be treated by private dental practitioners after agreements. In terms of employment, the PDS is the first employer for most newly graduated dentists, but after a few years, many of them move to the private dental sector. In recent years, Sweden has seen an expansion of the private dental sector, particularly in the larger cities, which has increased the competition for dentists between the public and the private dental care. Consequently, the PDS currently suffers from a shortage and high turnover of dentists due to difficulties in recruiting and retaining dentists [5].

However, the problem is not new. The PDS was introduced in 1938 and the difficulty in recruiting and retaining dentists has been reported since the 1940s. This was attributed to perceived unfavourable working conditions, such as high demands and low rewards, detailed control systems, and dentists’ lack of self-determination. Dentists in the PDS have in later decades reported similar experiences of a perceived lack of independence and encouragement of initiative-taking [6]. Moreover, according to a recent Swedish governmental report, the appeal of working in the Swedish PDS has diminished because of financial pressure, decreased degree of dental care for adults, and lower salary growth compared to the private dental sector [7]. Furthermore, general dentists in the PDS often perceive high work pressure [8, 9]. However, perceived dissatisfaction with working for the PDSs is not unique for Sweden. In the United Kingdom (UK), factors contributing to dissatisfaction include difficulties in providing quality dental care, and inadequate time to spend with patients [10]. In Finland, dental work is particularly demanding for dentists employed in the public sector [4].

To ensure a high quality of care and mitigate the staff shortage issue, chief dental officers of the Swedish PDS strive to attract dentists [11]. Fulfilling this ambition depends on the PDS organisations’ ability to stand out in the competition with the private dental sector. PDS organisations might also have to compete with each other to attract dentists. This study focuses on how the Swedish PDS organisations use their websites to attract dentists to work for them.

To attract potential employees and to stand out in the competition, organisations’ employer branding plays an important role. Employer branding concerns how organisations communicate about themselves as employers, how they differ from other organisations, and what makes them desirable to work for [12]. Healthcare organisations need to take employer branding seriously due to staff shortages [13]. The foundation of employer branding is that the attractiveness of an organisation is influenced by job seekers’ perceptions of job and organisational characteristics [12], such as salary, career opportunities, and location. It also depends on whether an organisation is seen as a trendy or prestigious place to work for [14]. Furthermore, companies that are perceived as good places tend to have development programmes, emphasise diversity initiatives, and encourage a fun work environment [15]. Desirable working conditions in healthcare organisations comprise meaningful work, competitive salaries, individual responsibility at work, work-life balance, collaboration, and opportunities for individual and career development. Introductions for newly hired employees, opportunities for research, and the availability of specialisation areas are also important to attract employees [7]. However, communicating such generally desirable working conditions in their branding does not enable organisations to differentiate themselves from their competitors. Moreover, organisations may struggle to stand out because jobs and organisations within a field of work are often quite similar [14].

Since an organisation’s attractiveness is essential to appeal to potential employees [16], the competition may motivate organisations to exaggerate the presentation of their positive attributes. The term ‘washing’ is commonly used to describe a gap between reality and empty promises [17, 18]; it indicates presenting misleading information, such as feigning ethical behaviour, aimed to divert attention from negative practices [19]. For example, ‘greenwashing’ refers to companies’ misleading communication about their environmental concerns, and ‘sportswashing’ describes aiming to improve the bad reputation of a country by building positive associations [20]. A study on the organisational use of social media to attract new employees used the concept of ‘career-washing’ to describe a dis-connection between an organisation’s promises of career opportunities and the employees’ lived experiences [17]. Furthermore, the term ‘job-washing’ refers to misleading communication about workplaces, where the best qualities of a job and the workplace are highlighted but the challenges are kept hidden [21]. However, organisational communication that does not purely reflect organisational practices can be seen as visions and ambitions for organisational improvements. Discrepancies between words and actions may have the potential to simulate organisational improvements [22].

Organisations can use different forms of communication to appeal to potential new employees, including their websites, social media, printed or online journals, and recruitment events. Digital channels, such as websites, have become a useful organisational tool for attracting potential employees [13]. Given the PDS’s need to attract dentists and on reports of dentists’ perceptions of unfavourable working conditions in the PDS, this study investigates how the PDS organisations present themselves as employers on their websites.

## Method

### Data collection

The empirical data were collected in June 2024 from the websites of the 21 Swedish PDS organisations. If there was no specific website for the PDS in a region, information was retrieved about the PDS from the website concerning the healthcare region as a whole. The data comprised of publicly available written information directed at individuals who might be seeking jobs. It concerned information regarding organisations as employers and their work environment. The information was collected from website sections titled ‘To work in the PDS in region X’, ‘Work with us’, ‘How it is to work with us’, and ‘Work and career’. The collected information was copied into a word document that was used in the data analysis. Information about regulated benefits (such as vacation, pension, and occupational healthcare) was not included.

### Data analysis

The data were analysed using qualitative content analysis, which is a suitable method for a subjective interpretation of the content of textual data through a classification process of coding and identifying themes or patterns [23]. The text was read thoroughly several times to get an overview, and during this process, it was coded and then organised into categories. Thereafter, these derived categories were grouped into more overarching themes based on their similarities and differences. In this study, preconceived categories were avoided because the aim was to describe the content through an inductive approach. With such an approach, the categories and their names were derived from the data during the analysis [23]. This does not mean that the interpretation was done from scratch. Literature on working conditions and organisational communication served as inspiration to discover patterns in the data. In other words, this process was not a linear reading and sorting of the text but an iterative process that oscillated between reading and interpreting the data [24].

No ethical committee approval was required for the study as the websites are publicly available.

## Results

Two themes were derived from the analysis: *fostering a sense of belonging* and *encouraging development.* The themes and the categories are detailed in Table 1.

**Table 1:** The derived themes and categories.

Themes	Categories
Fostering a sense of belonging	Introduction for new dentists
	Appreciation of dentists
	Collaboration opportunities
Encouraging development	Education, growth, and career paths
	Transition support for newly graduated dentists
	Mutual investment

### Fostering a sense of belonging

This theme examined how new dentists were welcomed to an organisation through an introduction programme, were shown appreciation, and were given opportunities to collaborate with others.

#### Introduction for new dentists

The website information stressed the importance of introducing and welcoming newly hired dentists to an organisation and described how the introduction could be carried out. The aims of introducing and welcoming new dentists differed. One PDS used introductions as a means to foster a sense of belonging and to allow dentists to thrive.

We believe in a clear introduction for you who start working for us in the PDS. We would like to get to know you. And for you to thrive, we believe it is important that you feel like a part of us. Therefore, we attach great importance to giving you as a new employee with us a good introduction. (Jönköping)

The introduction was also a means to make dentists feel comfortable at the new workplace, to adjust to the needs of the individual dentist, and to create room for growth.

When you are new to the job, you should get the security you need. That is why we have an individual introduction and mentoring program that will give you a good foundation for your future development with us. Regardless of whether you are a recent graduate or have a few years of experience in the profession, we will give you room to grow. (Gävleborg)

Another aim was to introduce the missions and organisational structure of a PDS to new dentists and other dental staff. The introduction was also connected to dentists’ work with colleagues and treatment of patients. For instance, it was described as a way to give new dentists the opportunity to be introduced to colleagues and social networks in the organisation, and to discuss patient cases. It could also comprise lectures by specialist dentists who would present new dental knowledge. One PDS organisation offered dentists support and mentoring as long as needed.

If you start working for us, an introduction and mentoring will start immediately and go on as long as needed. If you have no or little work experience, the need for mentoring and good advice are especially welcomed. (Sörmland)

#### Appreciation of dentists

PDS organisations stressed that dentists who worked for them will have the opportunity to make a difference in their workplaces and to influence their work situation. For example, one PDS stated, ‘Here, you are given the space to influence, be seen, and make a difference’ (Blekinge). Another PDS organisation emphasised, ‘At the PDS, we actively work to ensure that everyone feels seen and is involved’ (Uppsala).

One organisation stressed that it was small and familiar, which was stated to indicate that there were ‘great opportunities to influence your work situation and career’ (Värmland). Similarly, another organisation emphasised that it was characterised by short decision paths and that dentists who worked for it will be respected and valued for their contributions.

Working for us means short decision-making paths and that you as an individual is seen as an individual and can make your voice heard. Here, you make an impact, and your engagement makes a difference. (Halland)

#### Collaboration opportunities

The PDS organisations stressed the availability of opportunities for dentists to collaborate with others at the workplace or within the organisation in question. Moreover, they highlighted the advantages of being part of a collective and described social relationships between dentists as a source of knowledge development, which in turn was supposed to lead to good working conditions and to create job satisfaction.

With us, you are an important part of the team. With hundreds of colleagues around the region, you have the opportunity to exchange and share knowledge in your daily work. Through our different experiences and roles, we will develop and learn from each other – it creates job satisfaction. (Norrbotten)

Social relationships at work were also described as a source of fun: ‘We have a lot of fun together’ (Östergötland). Furthermore, one organisation described itself as a ‘family’, using the word as a metaphor for a caring atmosphere, and emphasised the beneficial effects of a familiar work environment.

We are like a big family where we take care of each other and help one another in the best way possible. We do this because we are convinced that it creates personal satisfaction and satisfaction. When we meet each other with warmth, humor, and compassion, we all feel good. (Sörmland)

### Encouraging development

This theme focused on how the organisations promoted skill development and growth opportunities, offered clear career paths, and supported the transition of newly graduated dentists into practice. The investment in dentists’ development was stressed as an organisational advantage.

#### Education, growth, and career paths

The organisations emphasised that they provided opportunities for growth and development for dentists. For instance, one PDS organisation stated, ‘We believe in competence development’ (Blekinge). It also stressed that the opportunities it provided were particularly good. Moreover, one PDS organisation capitalised on its size to highlight its broad range of knowledge and the possibility for dentists to grow and develop together with others.

With us, you have great opportunities to grow and develop alongside many colleagues. Our size and broad and deep knowledge enable us to provide our employees with the opportunity to grow and develop. (Stockholm)

Some organisations pointed out that all dentists, irrespective of work experience, need further education and offered opportunities for them to continue training throughout their working life. The focus on the development and growth opportunities concerned not only the dentists’ professional role and dental practice but also their personal development. Regarding dental practice, the learning and development opportunities included mentorship, courses, education programmes, and training at specialist dental clinics. Some organisations also offered research opportunities or education programmes leading to management positions. In other words, dentists who wanted to grow and develop could have access to different activities and career paths if they joined the PDS. One organisation stated,

With us, you get the chance to grow throughout your career and become your best professional self. (Skåne)

#### Transition support for newly graduated dentists

In addition to providing introductions to newly hired dentists (as previously mentioned), the organisations paid special attention to introducing newly graduated dentists to the workplace and to dental practice. The introduction for this group comprised of dental clinical training and theoretical lectures, and they were assigned mentors who supported the development of their professional confidence and competence in treating patients. The length of the introduction programmes varied between organisations; according to their websites, it typically lasted through the dentists’ first year of work, but it could also depend on the needs of the individual dentist.

It is important to us that you who are new to the profession get good mentoring so that you feel secure at the beginning of your career. You will get a mentor during your first year with us, or longer if needed. (Värmland)

One organisation pointed out that the PDS was a preferable first workplace for newly graduated dentists because of its ambition to transition new dentists into professional practice through providing supporting mentors. It also explicitly mentioned the importance of introductions in making the PDS an attractive employer.

The PDS is a great workplace for those who have recently completed their education. To ensure that the time with us is rewarding and educational, a mentor is appointed to help and support the new employee…. Introducing new employees is an important basis for the public dental services to be able to attract and recruit personnel. (Uppsala)

#### Mutual investment

The organisational opportunities for dentists to develop their competence and grow were not provided without motives. As stated by one organisation, ‘If you invest in us, we will invest in you’ (Västernorrland). One organisational objective for investing in new dentists was to retain them; that is, providing opportunities for growth and development might motivate the dentist to stay.

It is important for us that you as an employee get a good start at your new job. Therefore, you are offered our one-year introduction program. Why? We want you to develop in your working role and gain high and broad competence. We want to keep you! (Norrbotten)

You have great opportunities to develop in your professional role at the PDS Västerbotten. Introduction and competence development begin on your first day of work. We want you to feel welcome and stay with us. (Västerbotten)

Another stated benefit for the organisations was making use of their dentists’ knowledge. For example, one organisation attributed its success to its employees: ‘It is our employees that make us successful’ (Stockholm). Another organisation stated, ‘If we are well educated and skilled, things will go well for us. Therefore, it is important that everyone has the opportunity to develop in our profession’ (Sörmland).

## Discussion

This study aimed to investigate how the Swedish PDS organisations presented themselves as employers on their websites. This focus was motivated by the fact that the Swedish PDS suffers from a shortage and high turnover of dentists, which requires PDS organisations to be perceived as attractive employers.

### Working conditions

This study showed that PDS organisations appealed to dentists through emphasising their commitment to (1) fostering a sense of belonging within the workplace and (2) encouraging development and career growth. The qualities and benefits offered to dentists on the PDS websites reflect what constitutes good working conditions. Both a sense of belonging [25] and opportunities for development and career [26, 27] can influence healthcare professionals’, including dentists’, perception of a workplace as attractive, which can explain why PDS organisations emphasise them. The results also indicate a strong belief in the importance of these working conditions because their mention in the PDS organisations’ descriptions of themselves as employers were almost identical. This suggests that the individual PDS organisations have not built their own unique employer brands. As a result, job seekers will face difficulties in distinguishing one organisation from another, which makes it difficult for any of these organisations to stand out and weakens their competitive advantage in attracting job applicants [12]. In other words, there is room for the individual PDS organisations to develop a unique picture of themselves and highlight why dentists should work for them.

### Focus on new graduates

The results indicate that the Swedish PDS organisations strove to particularly appeal to newly graduated dentists. The content of their introduction programmes was adjusted to meet the needs of this group through introduction programmes and mentors. The organisations’ offer to new graduates seems to be in congruence with what this group of dentists appreciate. A study of dentists who graduated during 2019–2021 showed that possibilities for guidance, teamwork, and career development were perceived as positive working conditions at their current workplaces [28]. Appealing to newly graduated dentists is in line with research suggesting that organisations ought to pay particular attention to young professionals as they need to know how these organisations will take care of them. Therefore, to be attractive, employers should listen to young professionals’ expectations and respond to them [13].

### Workforce turnover

Although this study focuses on the recruitment of dentists, the PDS organisations’ communication about themselves as employers will also be discussed in relation to the retainment of dentists. The possibility for these organisations to succeed in their ambition depends on dentists’ experiences of working for them. The PDS competes with the private dental sector, and the PDS organisations in the 21 regions also compete with each other. Discrepancies between how the organisations present themselves on their websites and how the dentists’ experience working for them may lead to dentists choosing to leave. Currently, national reports indicate that the turnover rate is high in the PDS due to perceived unfavourable working conditions [5, 7]. Another reason for the high turnover may be that the PDS is the first employer for many dentists, who may view the public dental sector as a place to develop their competence before they work as private dental practitioners, get a doctorate, or undergo specialty training [29]. The movement of dentists from the PDS to the private dental sector may also be attributed to dentists identifying more with their profession than with the organisation they work for [30]; consequently, they have no loyalty to the organisation they work for and see PDS organisations as suitable places to practice their work and gain experience [31]. Dentists may also switch employers as a way to increase their salaries.

The PDS organisations stressed the importance of motivating dentists to stay and work for them through welcoming workplaces and development opportunities. They also associated quality dental work with well-educated dentists. This should be interpreted in relation to the tendency of dentists to leave the Swedish PDS to work in the private sector, implying that there are few remaining experienced dentists who can provide all kinds of treatments in the PDS [7]. Vacancies also have consequences for access to dental care, such as prolonged patient waiting time, which might impact the oral health of the population. Moreover, the high turnover of junior dentists can have a negative impact on the productivity of the public dental sector and increase the strain on the remaining senior dentists, who are expected to mentor and support newly graduated dentists [32]. None of the PDS organisations mentioned the risk of heavy workload because of the lack of dentists and, therefore, high patient pressure. Further, the opportunities for dentists to discuss and broaden their knowledge together with colleagues may be reduced due to the high turnover of dentists in the PDS and the resulting workload. Therefore, it is important for public dental organisations to be viewed as attractive workplaces, both to recruit and to retain dentists.

It stands to reason that PDS organisations present themselves in a favourable way, and that the working conditions might vary between the different PDS organisations and the different workplaces within the organisations. However, how employees perceive communicated promises of a job have an impact on the organisations’ ability to attract them. Perceived unfilled promises can lead to negative attitudes towards an organisation and a lack of loyalty [33]. Thus, if dentists perceive that the gap is too wide between what the PDS organisations communicate and how they perceive the actual working conditions, the attractiveness of the PDS is likely to remain lower than that of the private sector. On the other hand, the PDS organisations’ promises of good working conditions might not be empty. Organisations’ communication with the public can be interpreted as visions for the future [18]. Although the words do not fully reflect current organisational practices, communication about ideals has the potential to improve workplaces [22].

### Limitations

The findings of this study should be interpreted in the light of some limitations. First, this study focused only on the Swedish PDS organisations’ websites. Further research on how the PDS organisations use other means of communication – such as social media, printed or online journals, and recruitment events – would provide a deeper understanding of how they appeal to dentists. Second, research is needed on dentists’ perceptions of the PDS organisations’ websites and their significance in attracting dentists compared to other information channels. Third, as the PDS organisations suffer from a high turnover of dentists, their websites might depict idealised working conditions to attract dentists. Therefore, further research is needed on dentists’ experiences of working for the PDS to confirm whether the content on the websites aligns with actual workplace realities or is perceived as misleading. Fourth, the results may not directly be generalisable to other countries due to different dental care systems. Therefore, studies in other countries with similar problems in recruiting and retaining dentists are needed. Finally, the data were interpreted by the author, who has a professional background as a dentist. As in all forms of qualitative research, personal biases could influence the interpretation, and other researchers may have analysed the data differently [34]. However, a preunderstanding of a topic can enable a nuanced and in-depth analysis of the empirical material and thus be a strength [35].

### Significance and practical implications

Despite the limitations, this study contributes to the knowledge of how the Swedish PDS organisations use their websites to position themselves as attractive employers, which is an underexplored aspect of dental organisations’ recruitment strategy. Furthermore, a strength of this study is that it includes all Swedish PDS organisations’ websites. Document studies’ capability to analyse large bodies of data, such as websites content, makes the method useful for providing broad insights into a topic [36]. Document studies can also be complemented with other research methods, such as interviews, to get richer empirical data, and generate new relevant research questions [37].

As previously mentioned in the section on limitations, this study recommends further research on PDS organisations’ use of their websites to attract dentists and PDS dentists’ perceptions of the content on the websites compared to their lived work experiences. Such research might support PDS organisations in effectively appealing to dentists and making relevant organisational improvements to meet dentists’ work expectations.

## Conclusions

This study showed that the Swedish PDS organisations aimed to attract dentists through offering good working conditions on their websites and that they particularly appealed to newly graduated dentists. Regarding the PDS suffering from a high turnover of dentists, this study opens avenues for future research on the significance of the PDS organisations’ websites in attracting dentists to work for them and on dentists’ perceptions of the content on the websites in relation to their work experiences, as unmet expectations might reduce dentists’ willingness to stay in an organisation.

## Conflicts of interest

The author has no conflict of interest.
